# Supplementary material for: A Critical View of the Application of the APEX Software (Aqueous Photochemistry of Environmentally-Occurring Xenobiotics) to Predict Photoreaction Kinetics in Surface Freshwaters
Source: Molecules. 2019 Dec 18;25(1):9. doi: 10.3390/molecules25010009 (PMC7017383; doi:10.3390/molecules25010009)
Supplement: Supplementary file 1 [file molecules-25-00009-s001.zip › 3.2.4_gcc-4.4.0.zip!/3.2.4_gcc-4.4.0/3.2.4_gcc-4.4.0/doc/octave/html/interpreter/Diagonal-Matrix-Functions.html]

Diagonal Matrix Functions - Untitled


Next: Permutation Matrix Functions,
Up: Function Support


---

#### 20.3.1 Diagonal Matrix Functions

inv and pinv can be applied to a diagonal matrix, yielding again
a diagonal matrix. det will use an efficient straightforward calculation
when given a diagonal matrix, as well as cond.
The following mapper functions can be applied to a diagonal matrix
without converting it to a full one:
abs, real, imag, conj, sqrt.
A diagonal matrix can also be returned from the balance
and svd functions.
The sparse function will convert a diagonal matrix efficiently to a
sparse matrix.
